# Supplementary material for: Isochoric Supercooling Organ Preservation System
Source: Bioengineering (Basel). 2023 Aug 7;10(8):934. doi: 10.3390/bioengineering10080934 (PMC10451689; doi:10.3390/bioengineering10080934)
Supplement: Supplementary file 1 [file bioengineering-10-00934-s001.zip › bioengineering-2473977-supplementary.pdf]

Isochoric Supercooling Organ Preservation System

SUPPLEMENTAL INFORMATION

Drawing sketches with dimensions

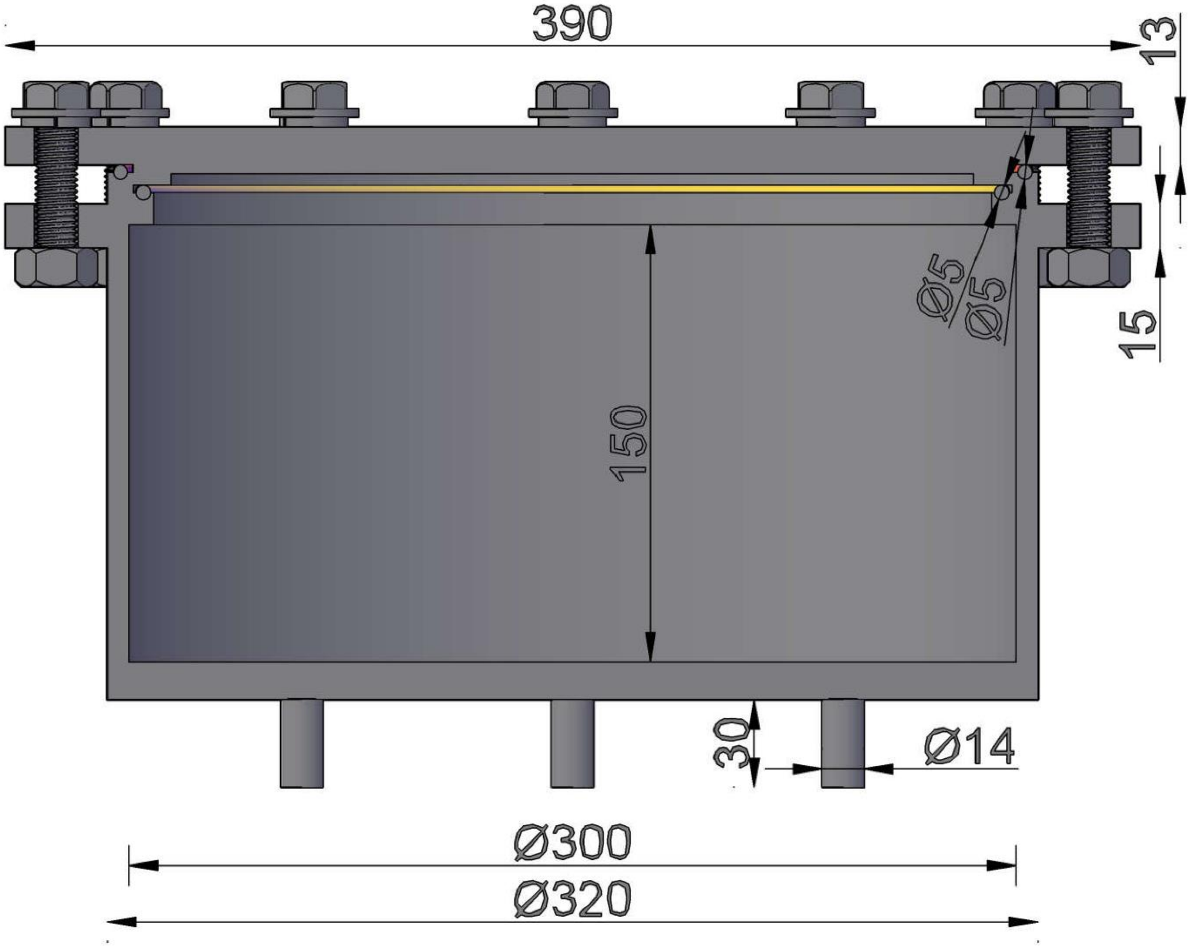

Figure S1. Section view of the isochoric chamber with dimensions.

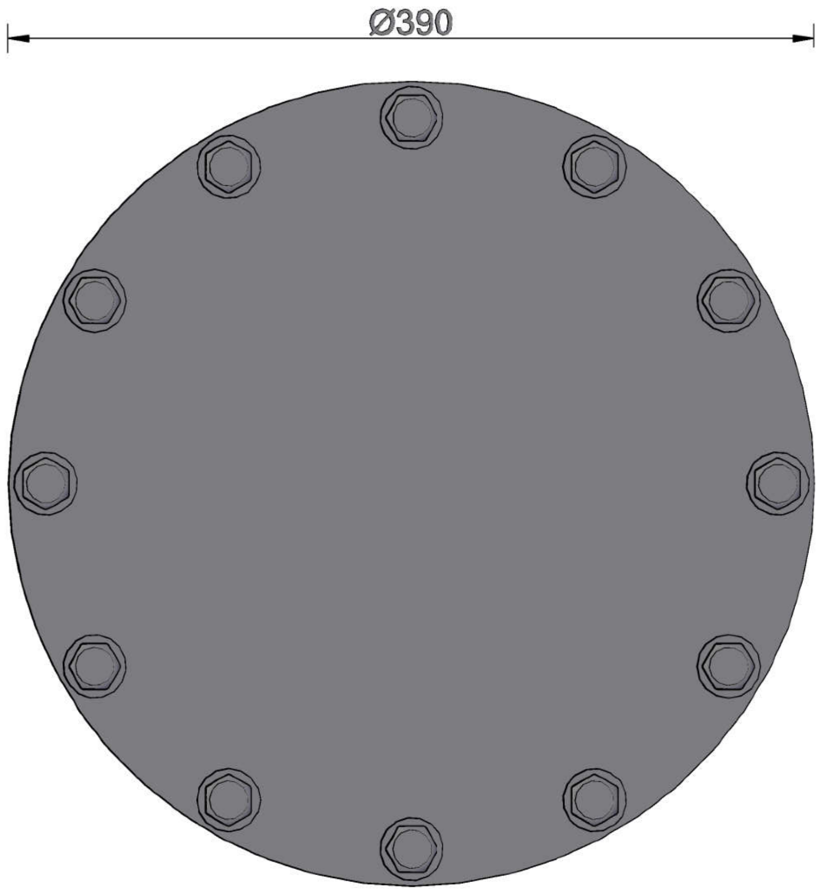

Figure S2. Top view of the lid with dimensions.

A detailed electric circuit is shown bellow

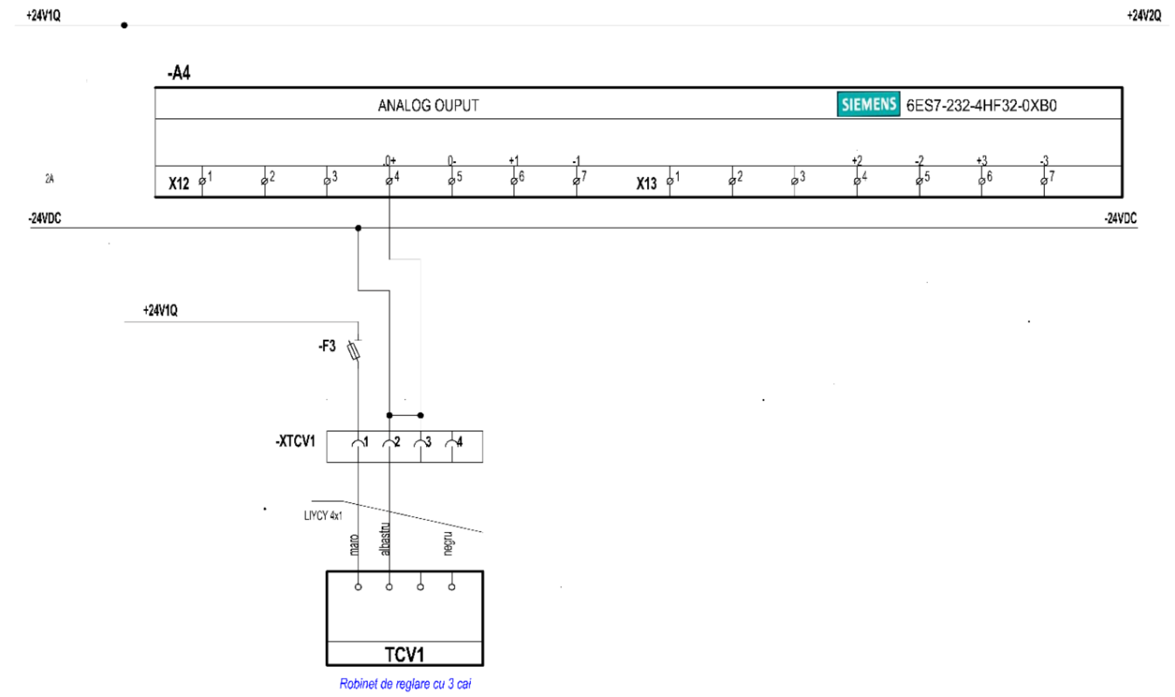

Figure S3. Analog outputs

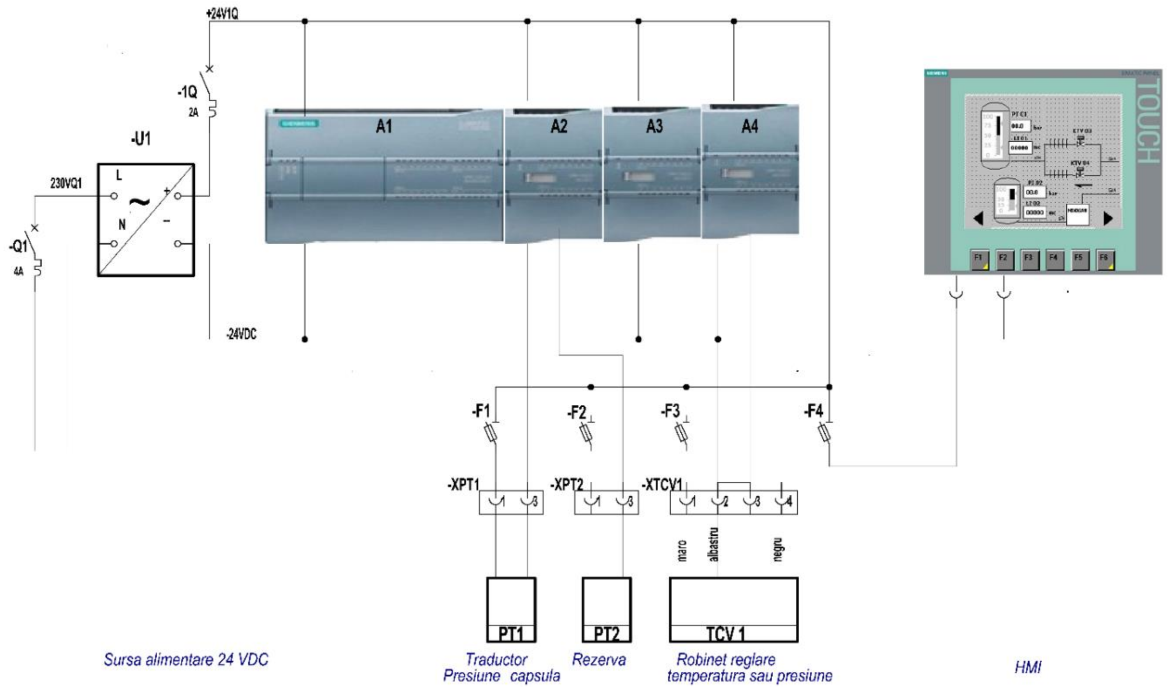

Figure S4. Power supply 230VAC, power supply 24VDC and PLC.

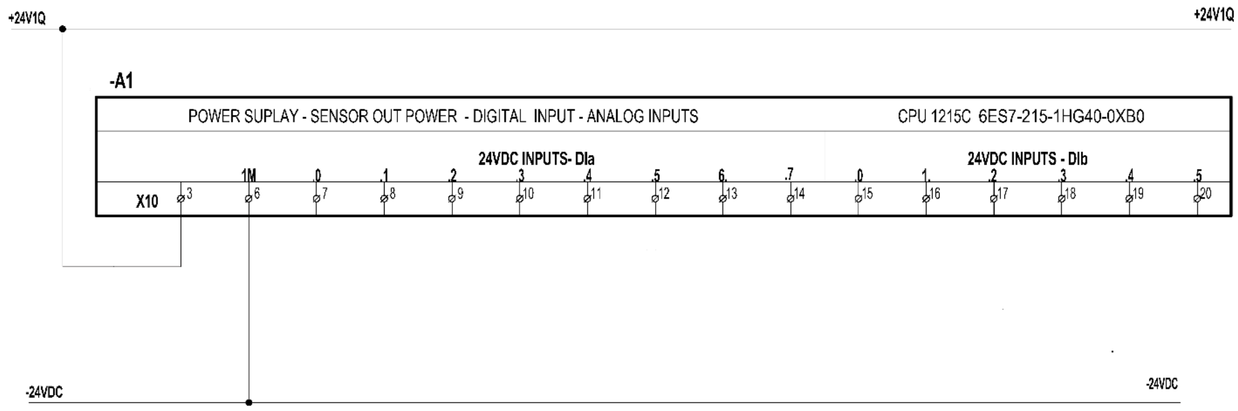

Figure S5. Digital inputs.

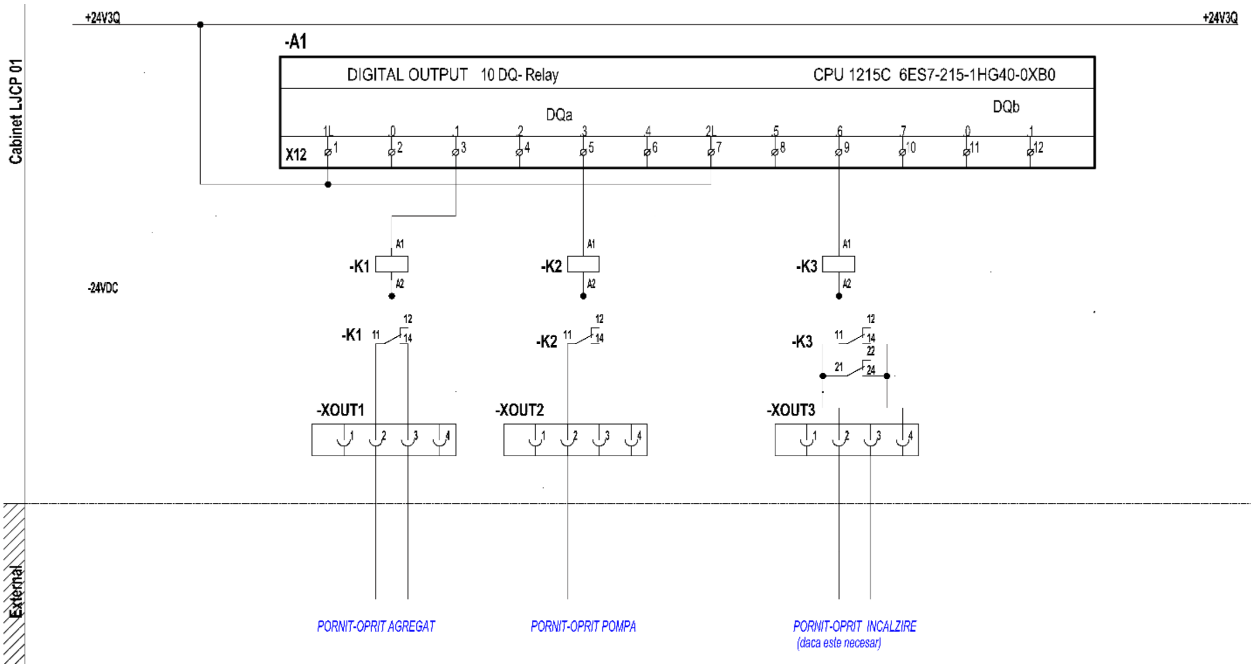

Figure S6. CPU – Digital outputs.

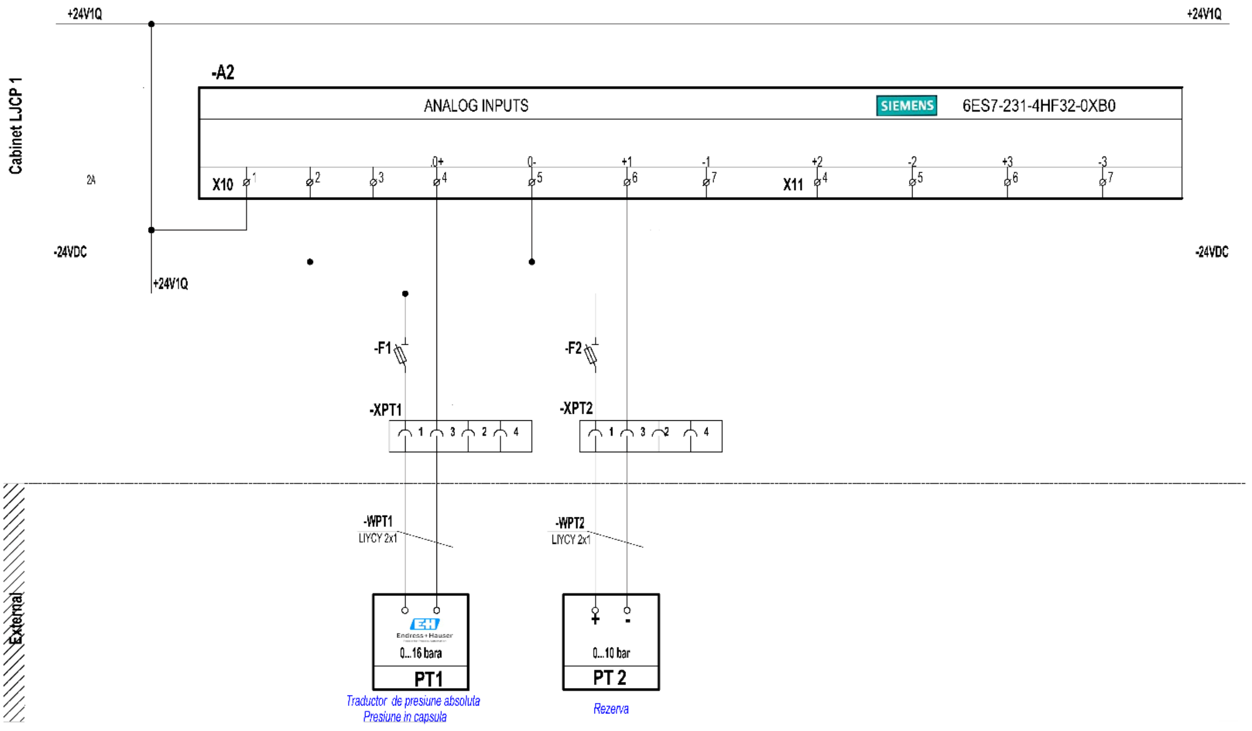

Figure S7. Analog inputs.

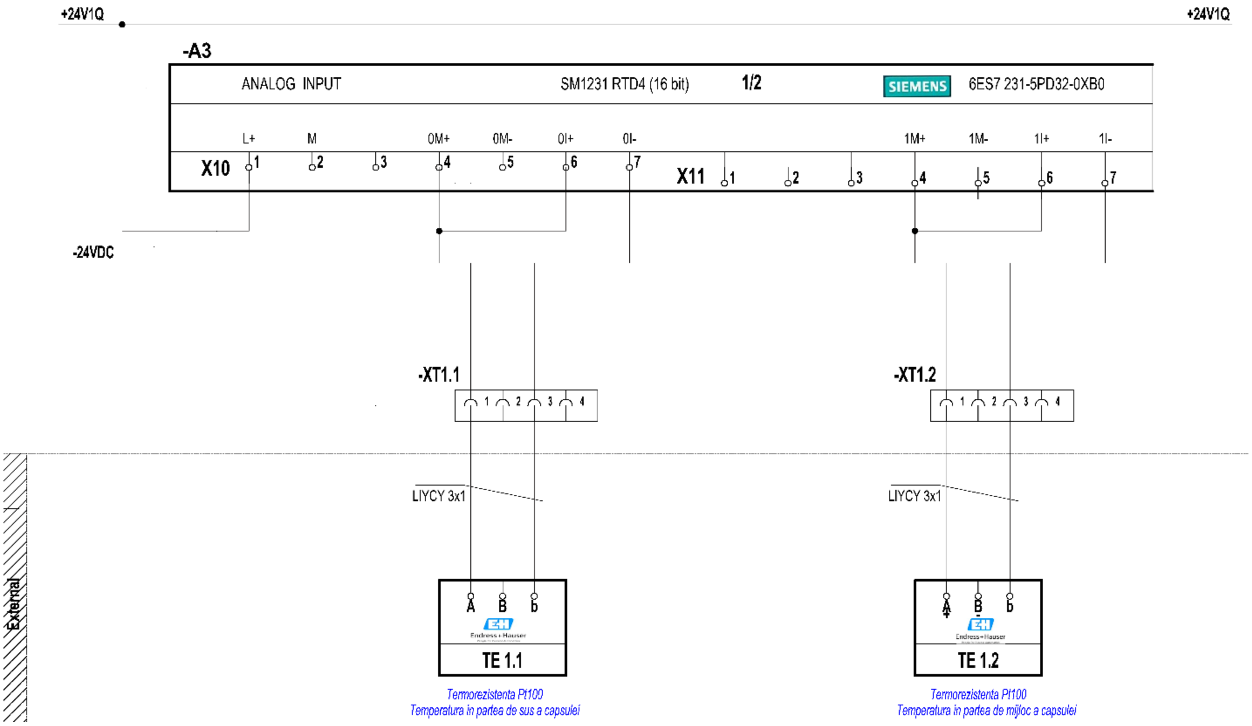

Figure S8. Analog inputs RTD.

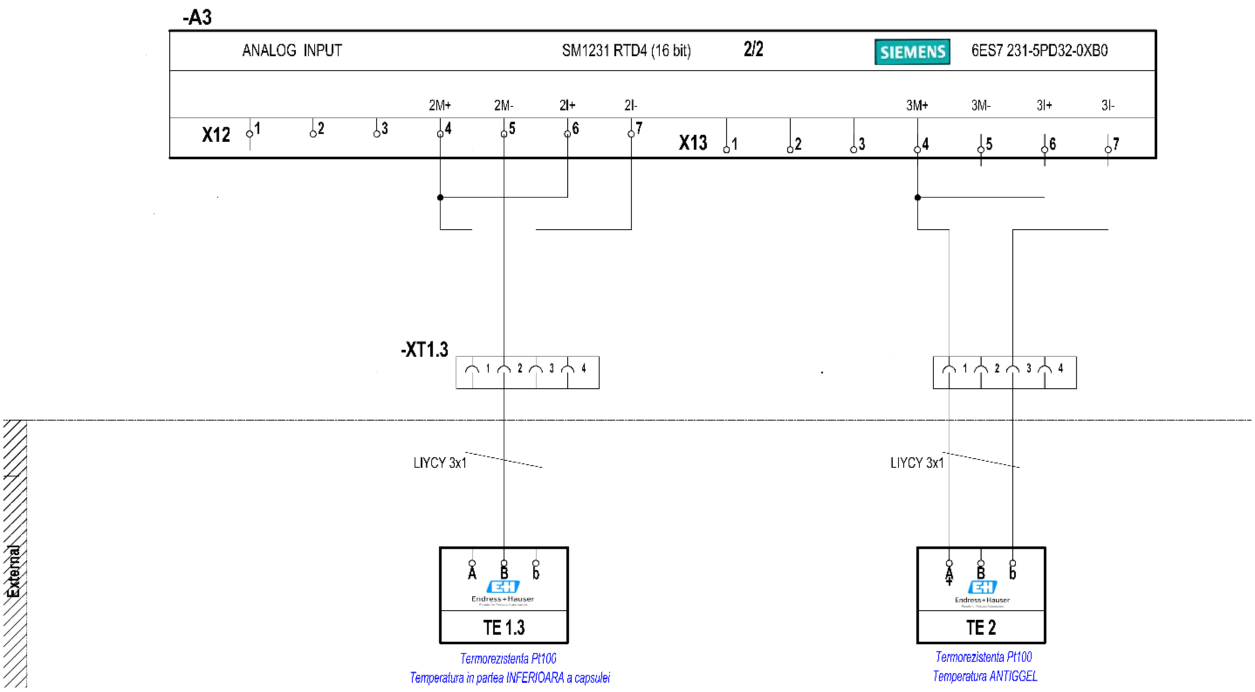

Figure S9. Analog inputs RTD.

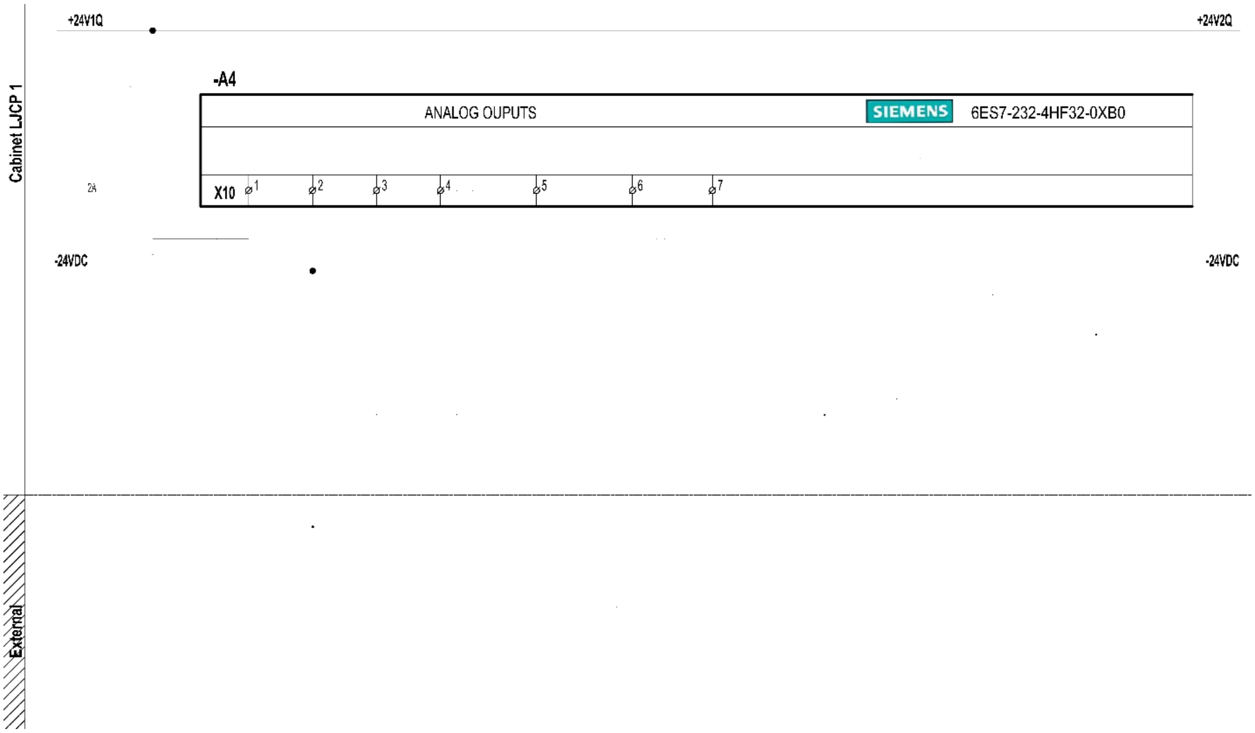

Figure S10. Analog outputs.
